# Numerical Study on Heat Transfer Characteristics of Microchannel with Ferrofluid Under Influence of Magnetic Intensity

**DOI:** 10.3390/mi17030383

**Published:** 2026-03-21

**Authors:** Seong-Guk Hwang, Tai Duc Le, Moo-Yeon Lee

**Affiliations:** Department of Mechanical Engineering, Dong-A University, 37 Nakdong-Daero 550, Saha-gu, Busan 49315, Republic of Korea; 2178735@donga.ac.kr (S.-G.H.); 2377988@donga.ac.kr (T.D.L.)

**Keywords:** battery thermal management, ferrofluid, magnetic intensity, magnetohydrodynamic (MHD) pump, microchannel cooling

## Abstract

Effective thermal management is critical for high-power lithium-ion batteries to mitigate excessive heat generation and ensure operational reliability. Failure to maintain a uniform temperature distribution can lead to accelerated capacity fading and severe safety risks, such as thermal runaway. In this study, a ferrofluid-based magnetohydrodynamic (MHD) microchannel cooling system was numerically investigated to elucidate the influence of magnetic intensity, magnet geometry, and electrical boundary conditions on flow behavior and heat transfer performance for battery cooling applications. A fully coupled multiphysics model incorporating electromagnetic, fluid flow, and heat transfer phenomena was developed and validated against experimental and numerical data from the literature. The results show that increasing the applied voltage enhances current density and Lorentz force almost linearly, leading to significant flow acceleration and improved convective heat transfer. Electrical insulation effectively suppresses current leakage into the channel walls, increasing the average current density by up to 222% and the Lorentz force by more than 300%. Compared with a cylindrical magnet, a rectangular magnet provides a more uniform magnetic field distribution and stronger near-wall Lorentz forcing, resulting in superior cooling performance. Under a 4C discharge condition, the insulated rectangular magnet reduces the maximum battery temperature by approximately 30% and increases the average Nusselt number by up to 103% relative to the non-insulated case. The findings reveal the critical roles of magnetic-field-controlled flow symmetry and near-wall forcing in MHD-driven microchannels, and provide practical design guidelines for battery cooling systems with no moving mechanical parts and active electromagnetic flow control.

## 1. Introduction

The rapid growth of high-power lithium-ion batteries in electric vehicles and energy storage systems has intensified demand for efficient, reliable thermal management solutions [1,2,3]. Excessive temperature rise and non-uniform temperature distribution within battery packs can accelerate degradation, reduce capacity and pose serious safety risks [4,5,6]. Consequently, advanced cooling technologies capable of providing high heat removal rates while maintaining structural simplicity and operational stability have attracted significant research attention [7,8].

Microchannel cooling has emerged as a promising approach due to its large surface-to-volume ratio and superior heat transfer performance [9,10,11]. However, conventional microchannel systems relying on mechanical pumps face challenges such as flow instability, noise, mechanical wear, and limited reliability at small scales [12,13,14]. To address these limitations, magnetohydrodynamic (MHD) pumping offers an attractive alternative. Specifically, MHD pumping drives electrically conductive fluids using the Lorentz force without moving parts [15,16,17]. The absence of mechanical components makes MHD pumps particularly suitable for compact, vibration-free battery-cooling systems [18,19,20].

Ferrofluids are colloidal suspensions of magnetic nanoparticles in a carrier liquid [21,22]. Ferrofluids exhibit unique thermomagnetic properties when subjected to external magnetic fields. Under magnetic influence, ferrofluids exhibit changes in flow structure, viscosity and thermal transport [23,24,25]. These characteristics enable ferrofluids to actively control heat transfer performance. The interaction among magnetic intensity, induced Lorentz forces, and ferrohydrodynamic effects gives rise to complex, coupled transport phenomena that can significantly enhance convective heat transfer in microchannels [26,27,28].

Recent numerical and experimental investigations have demonstrated that applying magnetic fields to ferrofluid flows in microchannels can significantly enhance convective heat transfer [29]. Subsequent studies demonstrated this enhancement primarily to magnetically induced vortices, recirculation zones, and boundary-layer thinning. For example, Marzougui et al. showed that increasing the magnetic intensity and Reynolds number promotes the formation of localized vortical structures near the magnet locations, thereby improving the average Nusselt number [30]. Similar mechanisms were reported in corrugated and curved minichannels, where the magnetic field acts as a “virtual obstacle”. These characteristics enhance mixing and produce downstream peaks in the Nusselt number distributions [31]. In addition, non-uniform magnetic fields combined with passive geometric features such as ribs further intensify secondary flows, yielding heat transfer improvements of up to 65% and significantly reduced pumping power requirements [32,33].

In parallel with externally magnetized flows, MHD pump-driven cooling systems have been explored as an alternative to mechanical pumping, particularly for high-power and reliability-critical applications. Hartl et al. [34] proposed a MHD liquid-metal cooling concept integrated into structural components and performed a comprehensive multiphysics modeling and parameterized optimization study. The results demonstrated that Lorentz-force-induced flow could effectively replace mechanical pumping in extreme thermal environments [34]. Experimental studies further confirmed the practical advantages of MHD pumping. Fan et al. reported device junction temperatures comparable to water cooling at substantially lower pump power and noise [35], while Yerasimou et al. showed that adaptive MHD actuation could actively suppress junction temperature fluctuations over load cycles [36]. More recently, MHD pump-based microchannel studies quantified the influence of applied voltage, Hartmann number, and channel cross-section on current density, Lorentz force, velocity and heat removal, establishing key electromagnetic–hydrodynamic performance relationships. Hwang et al. developed a coupled multiphysics model to evaluate an MHD pump-based cooling system for a 22 kW EV traction inverter. The results showed that the MHD pump provides self-circulation of the ferrofluid, enabling higher outlet velocity and improved convection. The study further reports that increasing the voltage and magnetic field strength strengthens the Lorentz-force-driven flow and enhances heat removal in ferrofluid [37]. Seo et al. [38] numerically studied an MHD pump-driven microchannel cooling system for a heat dissipating element using a coupled multiphysics model. Pump performance was quantified via current density, magnetic flux density, Lorentz force, shear stress, and velocity by varying applied voltage and Hartmann number (Ha). The results demonstrated that increasing voltage and Ha reduced the maximum heater temperature and increased heat removal. Specifically, heat removal increased by 34.5% when the voltage rose from 0.05 to 0.35 V at a Ha of 2.0, and by 39.5% when Ha increased from 1.41 to 3.76 at 0.05 V [38]. Hasan et al. [39] numerically investigated how the microchannel cross-section geometry affects the performance of the MHD pump. The results indicated that the velocity and flow rate increased with increases in both the current and magnetic field due to increased Lorentz forcing. In addition, for equal pump volume, the circular channel produced the highest velocity, while trapezoidal channels were most penalized by corner regions [39].

Previous studies have investigated ferrofluid heat transfer and MHD pumping mechanisms in several contexts. Early work mainly focused on the fundamental thermo-fluid behavior of ferrofluids in simplified geometries such as square or rectangular cavities under external magnetic fields, where the influence of magnetic intensity, viscosity, and nanoparticle concentration on natural convection heat transfer was analyzed. More recent research has extended these concepts to microscale cooling systems using MHD pumps to circulate conductive coolants through microchannels for generic heat dissipating elements or power electronic components [37,38]. However, despite these advances, the integration of ferrofluid-based MHD cooling with battery thermal management systems remains insufficiently explored. In particular, the coupled effects of ferrofluid electrical conductivity, current-driven Lorentz forces, magnetic fields, and realistic boundary conditions relevant to battery modules have not been systematically investigated.

In addition, several technical challenges arise when modeling ferrofluid-based MHD cooling systems. These include ensuring numerical stability for the strongly coupled electromagnetic-fluid-thermal governing equations, defining appropriate electrical boundary conditions for current injection and potential distribution, and representing realistic electrical conductivity of the Fe_3_O_4_/water ferrofluid used in the MHD model. To address these challenges, a fully coupled multiphysics formulation was implemented in which the electric current, magnetic field, fluid flow, and heat transfer equations were solved simultaneously. Furthermore, careful specification of the material properties and boundary conditions was adopted to ensure numerical convergence and physical consistency of the predicted Lorentz-force-driven flow and heat transfer behavior.

Therefore, the objective of the present study was to numerically investigate the performance of a ferrofluid-based MHD cooling system for battery thermal management using a coupled electromagnetic-fluid-thermal model. The study developed a fully coupled multiphysics framework to analyze electromagnetic-driven ferrofluid flow and heat transfer in a battery cooling configuration. It further examines how magnet geometry and electrical boundary conditions influence current distribution and Lorentz force generation in the cooling channel. Based on these analyses, the resulting flow acceleration and convective heat transfer enhancement were evaluated in the context of practical battery thermal management applications. These results provide insights into the feasibility and design considerations of MHD-driven cooling for battery systems.

## 2. Method

### 2.1. Numerical Modeling

Numerical simulations were conducted using COMSOL Multiphysics 6.1, employing the AC/DC, fluid flow, and heat transfer physics. The numerical model developed in this study represents a ferrofluid-based MHD microchannel cooling system applied to a lithium-ion battery. A LiFePO_4_ pouch cell was considered as the heat source. The geometric dimensions and material properties of the battery are listed in Table 1. The total heat generation rates of the lithium-ion pouch cell were reported as 19.4 W, 36.9 W, 56.2 W, and 82.3 W for the 1C, 2C, 3C, and 4C discharge rates, respectively, based on experimental measurements reported by Panchal et al. [40]. These values were converted to volumetric heat generation rates for use in the numerical model by dividing the total heat generation by the battery volume. This conversion allows the heat source term in the energy equation to be implemented as a uniform volumetric heat generation term within the battery domain. In the present numerical model, the volumetric heat generation rate within the battery was assumed to be constant for each discharge condition. This assumption was based on the steady-state formulation used in the simulations and on the experimentally reported heat generation values for LiFePO_4_ pouch cells operating at different C-rates. For each discharge rate, the heat generation is prescribed as a uniform volumetric source term representing the average heat produced during steady discharge. Such an approach is commonly used in numerical investigations of battery thermal management systems, where the primary objective is to evaluate the effectiveness of the cooling strategy rather than to model detailed electrochemical heat generation mechanisms.

A microchannel cooling plate was positioned beneath the battery cell to dissipate the generated heat. The cooling plate consisted of 10 parallel fins, forming uniform flow passages to enhance convective heat transfer from the battery surface. To eliminate mechanical pumping components, an MHD pumping section was integrated upstream of the microchannel, enabling flow generation through electromagnetic forcing.

The MHD section comprised permanent magnets and electrodes, with an optional electrical insulation layer applied selectively to the channel walls. Specifically, an electrically insulating rubber layer was applied along the side walls of the microchannel adjacent to the electrode region to prevent current leakage into the battery structure. The layer thickness was t = 5 mm. The rubber was modeled as an electrical insulator with σ ≈ 10^−12^ S/m and thermal conductivity k ≈ 0.5 W/m·K. The insulating layer was positioned between the electrode structure and the external solid boundary, and did not lie along the primary heat transfer path from the battery to the coolant channel. Therefore, its influence on the battery to coolant thermal resistance was negligible in the present configuration. The channel geometry and hydraulic cross-section remained unchanged.

**Figure 1:**
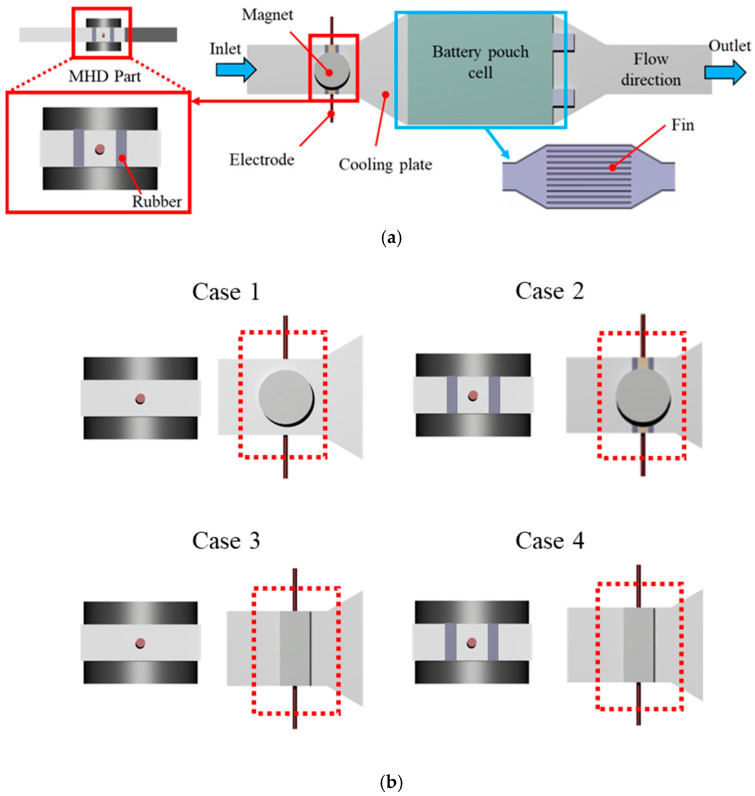
Schematic of the MHD cooling system: (**a**) overall system configuration and (**b**) design cases of the MHD pump in the present study.

Based on magnet geometry and electrical boundary conditions, four design configurations were examined, combining cylindrical and rectangular magnets with electrically insulated and non-insulated walls. A schematic illustration of the overall system and design cases is shown in Figure 1. The detailed geometries and dimensions of the battery, magnets, electrodes and cooling plate are summarized in Table 1. In addition, a detailed description of the different configurations of the MHD system in the current study is illustrated in Table 2.

A Fe_3_O_4_-based ferrofluid was employed as the working fluid to investigate magnetically induced flow and heat transfer behavior. The ferrofluid consists of magnetic nanoparticles uniformly dispersed in water. The macroscopic properties of ferrofluid are strongly influenced by the nanoparticle volume fraction. In the present study, the nanoparticle volume fraction was fixed at 10% to enable a systematic comparison of magnetic intensity and magnet configuration effects under consistent thermophysical conditions.

The effective thermophysical and electrical properties of the ferrofluid were evaluated using widely adopted mixture correlations reported in the literature. The effective density, dynamic viscosity, volumetric heat capacity, thermal conductivity, and electrical conductivity were defined as functions of the nanoparticle volume fraction, assuming homogeneous dispersion and thermal equilibrium between the solid and liquid phases.

The effective density of the ferrofluid is calculated as the volumetric average of the base fluid and nanoparticles [41]:

(1) $$\rho_{nf}=(1-\phi )\rho_{f}+\phi \rho_{p}$$

The effective dynamic viscosity accounting for the nanoparticle volume fraction is defined as [41]:

(2) $$\mu_{nf}=\mu_{f}(1+2.5\phi +6.5\phi^{2})$$

The effective volumetric heat capacity of the ferrofluid is given by [42]:

(3) $$(\rho C_{p}{)}_{nf}=(1-\phi )(\rho C_{p}{)}_{f}+\phi (\rho C_{p}{)}_{p}$$

The effective thermal conductivity is evaluated using a mixture model that considers the thermal interaction between the nanoparticles and the base fluid [42]:

(4) $$k_{nf}=k_{f}[\frac{\left(k_{p}+2k_{f})-2\phi (k_{f}-k_{p}\right)}{\left(k_{p}+2k_{f})+\phi (k_{f}-k_{p}\right)}]$$

Finally, the effective electrical conductivity of the ferrofluid is calculated using a linear mixture relation as [42]:

(5) $$\sigma_{nf}=(1-\phi )\sigma_{f}+\phi \sigma_{p}$$

where ϕ denotes the nanoparticle volume fraction, while the subscripts nf, f, and p represent the ferrofluid, base fluid, and nanoparticles, respectively.

In practical battery systems, supplying electric current through a conductive coolant requires careful electrical insulation and corrosion control. Electrolytic reactions, electrode degradation, and safety considerations must be addressed through insulated electrodes, controlled current levels, and chemically stable ferrofluid formulations. Therefore, the present study focused on the numerical evaluation of the MHD enhancement mechanism, while acknowledging that system integration requires further experimental investigation.

In the present study, the nanoparticle concentration ϕ denotes the volume fraction of Fe_3_O_4_ nanoparticles in the base fluid, not the weight fraction. The selected 10% volume fraction was adopted to examine the upper-bound performance of the ferrofluid-based MHD cooling configuration under fixed thermophysical conditions. The effective-property relations in Equations (1)–(5) were employed as first-order mixture approximations under the assumptions of homogeneous dispersion and thermal equilibrium between the solid and liquid phases. However, it is acknowledged that a concentration of 10% volume fraction is relatively high, and the applicability of dilute-suspension correlations becomes less rigorous at this level. In practical ferrofluids, particle aggregation, sedimentation, or field-induced chaining may occur, thereby modifying the effective properties of the suspension. In particular, such effects may increase the apparent viscosity, thereby increasing the flow resistance and reducing the Lorentz-force-driven velocity, while the effective electrical conductivity may deviate from the linear mixture estimate because particle clustering can alter conductive pathways. Therefore, the present model should be interpreted as a comparative numerical framework for evaluating the effects of magnetic intensity, magnet geometry, and electrical boundary conditions, whereas detailed stability characterization and experimental verification for highly concentrated ferrofluids remain important topics for future work. The thermal-physical properties and electrical conductivity of the base fluid, Fe_3_O_4_ nanoparticles, aluminum, rubber, and the battery used in the electric current model for the permanent magnet model are explicitly listed in Table 3 to ensure completeness of the MHD formulation and simulations.

In the present numerical simulations, the effective thermophysical and electrical properties of the ferrofluid were calculated using mixture models based on the nanoparticle volume fraction, as given in Equations (1)–(5). These correlations are widely adopted in numerical studies of ferrofluid heat transfer and magnetohydrodynamic flows under moderate temperature ranges. Since the cooling system’s operating temperature range in this study was relatively narrow, the thermophysical properties were assumed constant throughout the computational domain. This assumption allows for a clear evaluation of the effects of electromagnetic forcing, magnet geometry, and electrical boundary conditions on the flow and heat transfer characteristics. Similar constant property assumptions have been commonly adopted in previous numerical investigations of ferrofluid-based MHD cooling systems [37,38].

### 2.2. Governing Equations

The ferrofluid flow within the MHD-driven microchannel was assumed to be steady, laminar, and incompressible. The coupled transport phenomena were modeled by solving the continuity, momentum, electric current, and energy equations, accounting for electromagnetic, fluid, and thermal interactions.

The conservation of mass is governed by the continuity equation [45]:

(6) $$\nabla \cdot \vec{V}=0$$

The momentum transport is described by Navier–Stokes equations with an additional Lorentz force term to account for magnetohydrodynamic effects [46]:

(7) $$\rho (\vec{V}\cdot \nabla )\vec{V}=-\nabla P+\mu \nabla^{2}\vec{V}+{\vec{F}}_{L}$$

where $\vec{V}$ is the fluid velocity vector, P is the pressure, ρ is the fluid density, μ is the dynamic viscosity, and ${\vec{F}}_{L}$ denotes the Lorentz force per unit volume.

The Lorentz force arises from the interaction between the electric current density and the magnetic field and is defined as [46]:

(8) $${\vec{F}}_{L}=\vec{J}\times \vec{B}$$

where $\vec{J}$ is the electric current density vector and $\vec{B}$ is the magnetic flux density vector.

The electric field and current distribution were computed using the electric current conservation equation, assuming quasi-static MHD conditions [46]:

(9) $$\vec{J}=\sigma \left(\vec{E}+\vec{V}\times \vec{B}\right)$$

where σ is the electrical conductivity of the ferrofluid and $\vec{E}$ is the electric field vector. The electric field vector is defined from the electric potential as $\vec{E}=-\nabla \varphi$. Therefore, the current conservation equation is written as $\nabla \cdot \vec{J}=0$, which gives $\nabla \cdot [\sigma \left(-\nabla \varphi +\vec{V}\times \vec{B}\right)]=0$. Induced magnetic field effects were neglected due to the low magnetic Reynolds number typical of microchannel flows.

The thermal behavior of the system was governed by the energy equation [46]:

(10) $$\rho c_{p}\left(\vec{V}\cdot \nabla T\right)=k\nabla^{2}T+Q$$

where T is the temperature, $c_{p}$ is the specific heat capacity, k is the thermal conductivity, and Q represents the volumetric heat generation rate applied within the battery cell. This formulation enables a fully coupled analysis of electromagnetic forcing, flow symmetry, and convective heat transfer in the MHD-driven microchannel cooling system.

### 2.3. Boundary Conditions

In the present study, appropriate boundary conditions were prescribed for the fluid flow, electromagnetic field, and heat transfer to ensure a fully coupled and physically consistent MHD simulation. For the fluid flow, a no-slip condition was imposed on all solid walls of the microchannel and MHD pumping section. At the channel inlet, the fluid velocity was set to zero, allowing the flow to be generated solely by the Lorentz force induced in the MHD section. Specifically, at the channel inlet, the velocity was set to zero to represent a quiescent initial/inlet state, because the present system is a self-driven MHD pump without any externally imposed pressure gradient or mechanical pumping. Flow is generated solely by the Lorentz body force in the MHD section. The steady-state solution was obtained by numerically solving the fully coupled electric current, magnetic field, laminar flow, and heat transfer equations until convergence. Thus, the zero inlet velocity condition does not imply zero flow everywhere. Rather, it specifies that no forced inflow is imposed, and the final flow field is established entirely by electromagnetic pumping. In addition, a pressure outlet condition with zero gauge pressure was applied at the channel outlet. All walls were assumed to be hydraulically smooth.

For the electromagnetic field, a constant electric potential difference was applied between the electrode pairs to induce the electric field required for MHD actuation. The electrodes were modeled using terminal and ground boundary conditions. Specifically, a constant voltage was prescribed at the terminal electrode, while the opposing electrode was set to 0 V as the reference potential. Depending on the design configuration, the remaining channel walls were treated either as electrically insulated or electrically conductive boundaries. The magnetic field was generated using permanent magnets with fixed magnetization. In addition, magnetic insulation was applied at the external boundaries of the computational domain. The resulting magnetic flux density exhibited a spatially non-uniform distribution that depended on the magnet geometry. Therefore, both cylindrical and rectangular magnets were considered to systematically examine the influence of magnetic field distribution on the Lorentz-force-driven flow and heat transfer characteristics.

For the thermal field, a volumetric heat generation rate corresponding to the specified battery discharge C-rate was applied uniformly within the battery domain. Thermal continuity was enforced at all solid–fluid interfaces. Convective heat transfer was considered within the ferrofluid domain, while all external solid surfaces were assumed adiabatic, except for the battery-heating surface. The inlet fluid temperature was fixed at the ambient reference temperature of 25 °C.

These boundary conditions enabled the systematic evaluation of the effects of magnetic intensity, magnet geometry, and electrical insulation on flow symmetry, Lorentz-force-driven pumping behavior, and heat transfer characteristics within the MHD microchannel cooling system. Table 4 summarizes the boundary conditions used in the current numerical simulations.

### 2.4. Dimensionless Numbers

In order to quantitatively evaluate the flow and heat transfer characteristics, the Reynolds number (Re), Nusselt number (Nu), and Hartmann number (Ha) were employed.

The local heat transfer coefficient was calculated as [47]:

(11) $$h_{x}=\frac{Q-Q_{loss}}{A(T_{w,x}-T_{b,x})}$$

where Q is the applied heat input. Because the present study was entirely numerical, the heat transfer parameters were evaluated directly from the simulated thermal field. Heat dissipation to the surroundings was considered by imposing a natural convection boundary condition on the external surfaces, with a convective heat transfer coefficient of 5 W/m^2^·K representing cooling by ambient air. Thus, $Q_{loss}$ refers to the convective heat loss from the outer boundaries to the environment. In the present analysis, the wall temperature ($T_{w,x}$) was defined as the average temperature of the upper and lower channel walls, whereas bulk fluid temperature ($T_{b,x}$) was calculated as the average of the fluid inlet and outlet temperatures. The heat transfer area A was determined from the 3D microchannel wall geometry.

The bulk temperature was evaluated based on the energy balance as [47]:

(12) $$T_{b,x}=T_{i}+\frac{\left(Q-Q_{loss})A(x\right)}{\dot{m}C_{p}}$$

where $T_{i}$ is the inlet temperature, $\dot{m}$ is the mass flow rate, and $C_{p}$ is the specific heat capacity.

The local Nusselt number was then defined as [48]:

(13) $$Nu_{x}=\frac{h_{x}D_{h}}{k}$$

where $D_{h}$ denotes the hydraulic diameter and k is the thermal conductivity of the working fluid. The Nusselt number represents the ratio of convective to conductive heat transfer, and higher values indicate enhanced heat transfer performance.

The Reynolds number was calculated as [48]:

(14) $$Re=\frac{\rho UD_{h}}{\mu }$$

The Hartmann number, which characterizes the relative influence of electromagnetic forces compared to viscous forces, was defined as [49]:

(15) $$Ha=BL\sqrt{\frac{\sigma }{\mu }}$$

where B is the magnetic flux density applied to the fluid, L is the characteristic length, σ is the electrical conductivity, and μ is the dynamic viscosity.

### 2.5. Mesh Independence Test

**Figure 2:**
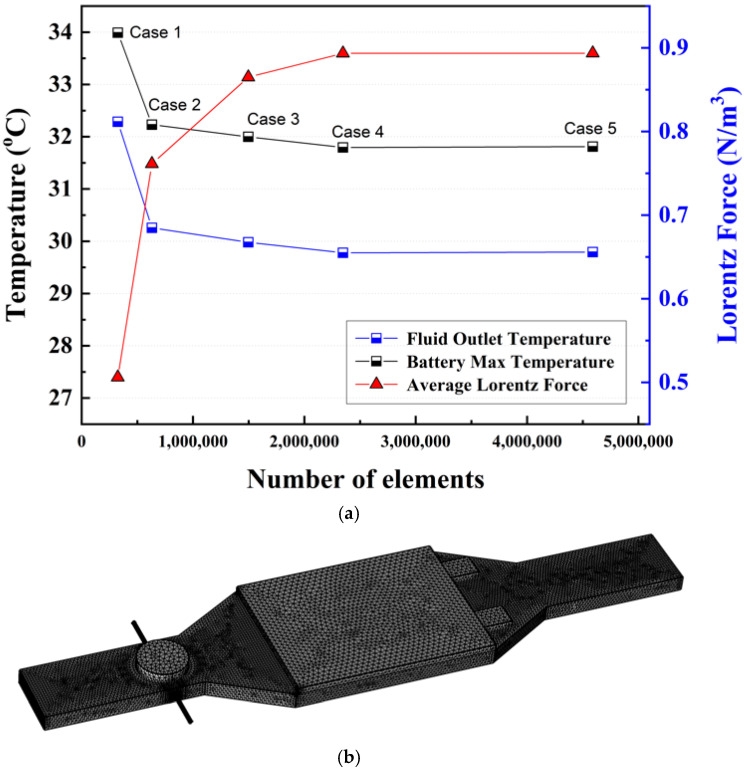
(**a**) Mesh independence test results and (**b**) mesh configurations utilized in the present study.

A mesh independence test was conducted to ensure the numerical reliability of the simulation results. Tetrahedral meshes were applied to the entire computational domain. Five different mesh configurations (Case 1–Case 5) with increasing mesh density were generated to examine the influence of mesh resolution on the numerical results. For each mesh configuration, the maximum battery temperature, outlet fluid temperature, and average Lorentz force were selected as representative parameters for comparison. The variations in these quantities with respect to the total number of mesh elements are shown in Figure 2a.

The results indicate that the differences between the mesh configurations of Cases 4 and 5 were negligible, with a maximum deviation of less than 0.06% in the battery maximum temperature and 0.017% in the average Lorentz force. However, the computational time required for Case 5 increased by more than 2 times that of Case 4 due to the significantly larger number of mesh elements. Considering both numerical accuracy and computational cost, the Case 4 mesh was selected as the optimal mesh configuration in this study. Accordingly, all subsequent simulations were performed using the Case 4 mesh. The mesh configuration applied in the numerical simulations is shown in Figure 2b.

## 3. Results and Discussion

This section systematically analyzes the electromagnetic behavior, flow characteristics, and cooling performance of the proposed MHD-assisted microchannel cooling system. In Section 3.1, the numerical model employed in this study is validated with previously reported experimental and numerical results. Section 3.2 investigates the electromagnetic characteristics of the MHD pump by quantitatively analyzing the magnetic field distribution, current density distribution, and Lorentz force under different magnet geometries and electrical boundary conditions. In Section 3.3, the influence of these electromagnetic characteristics on flow acceleration and flow structure is examined using velocity distributions and streamline analyses. Finally, Section 3.4 evaluates the cooling performance of the system by comparing the battery temperature, heat removal rate, and average Nusselt number under varying applied voltages, magnet geometries, and insulation conditions. These studies provide a quantitative assessment of the heat transfer enhancement achieved by the proposed MHD microchannel cooling system.

### 3.1. Validation

**Figure 3:**
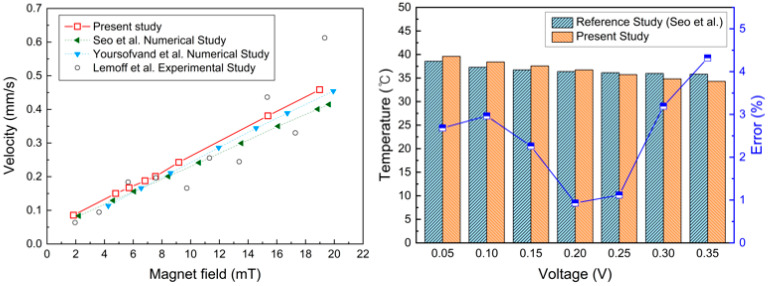
Validation of the numerical model [38,46,50].

To verify the reliability of the numerical model developed in this study, the simulation results were validated against previously reported experimental and numerical data available in the literature. The validation comparison is presented in Figure 3, using reference results obtained under magnetohydrodynamic (MHD) pump operating conditions. In earlier studies, the velocity and temperature distributions of MHD-driven cooling flows have been widely adopted as benchmark parameters for assessing numerical accuracy. Accordingly, the present validation focuses on flow behavior induced by the Lorentz force under an applied electric current of 15 mA and a magnetic field strength ranging from 2 to 20 mT.

The numerical results obtained in this study show excellent agreement with the experimental measurements reported by Lemoff et al. [50], particularly in capturing the trends in velocity variation associated with electromagnetic forcing. Under identical operating conditions, the maximum deviation between the present predictions and the experimental data was limited to 2.52%, while the average error remained below 1%, indicating high numerical accuracy. In addition, the predicted results closely followed the numerical trends reported by Seo et al. [38] for the same experimental dataset, further confirming the validity and robustness of the present numerical model. Therefore, the proposed numerical model was accepted for further simulations of the MHD microchannel cooling system.

In addition to validating the overall multiphysics model, the electromagnetic sub-model used to calculate the electric current density and Lorentz force was benchmarked against previously reported MHD micropump studies. Under quasi-static MHD conditions, the interaction between the electric current density and the magnetic flux density governs the generation of the Lorentz force that drives the flow. The present numerical predictions reproduced the expected linear relationships among applied voltage, current density, and the Lorentz force, consistent with theoretical MHD formulations and previously reported numerical results for MHD pump systems. Furthermore, the predicted flow acceleration and velocity distributions induced by Lorentz forcing showed good agreement with experimental observations reported by Lemoff et al. [50] and numerical investigations by Seo et al. [38], confirming the validity of the electromagnetic-fluid coupling approach adopted in the present study.

### 3.2. Electromagnetic Characteristics of MHD Pump

**Figure 4:**
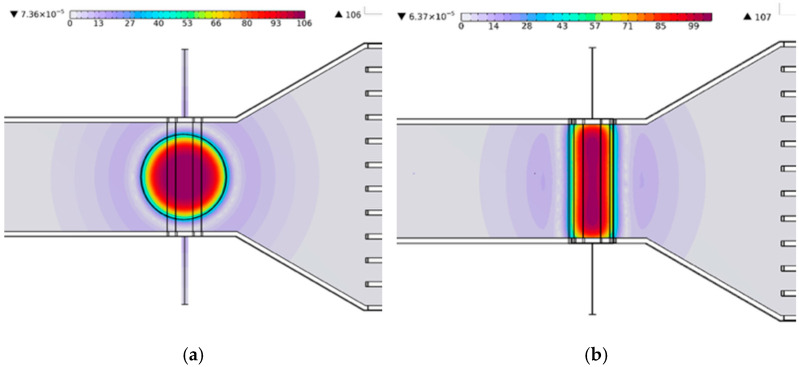
Normal magnetic flux density distributions: (**a**) cylindrical magnet and (**b**) rectangular magnet.

**Figure 5:**
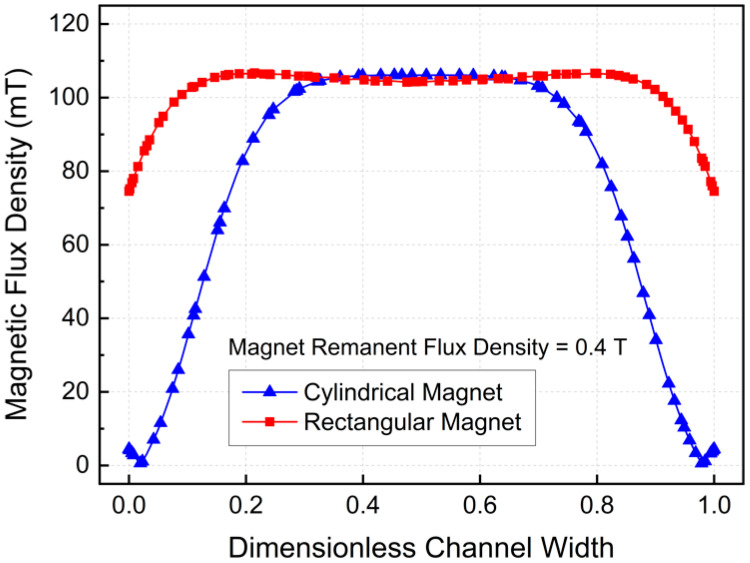
Magnetic flux density variation with dimensionless width.

Figure 4 and Figure 5 show the distributions of the normal magnetic flux density generated by the cylindrical and rectangular magnets in the MHD pumping region, illustrating the magnetic field acting on the ferrofluid along the channel thickness. To ensure a fair comparison of magnet geometry effects, both magnets were designed with identical volumes and equivalent effective cross-sectional areas normal to the magnetic field direction in contact with the channel. The remanent magnetic flux density was fixed at 0.4 T for both cases.

Under these conditions, the maximum magnetic flux density was nearly the same for the two configurations, reaching 106.1 mT for the cylindrical magnet and 106.7 mT for the rectangular magnet. However, the average magnetic flux density within the fluid domain differed significantly. The cylindrical magnet yielded an average value of 76.5 mT, whereas the rectangular magnet produced a substantially higher average of 102.56 mT, corresponding to an increase of approximately 34.06% compared to the cylindrical magnet. These results indicate a marked enhancement in magnetic field coverage for the rectangular geometry. This difference arises from the spatial distribution of the magnetic field. Specifically, the cylindrical magnet concentrates magnetic flux near the channel center, while the rectangular magnet generates a more uniformly distributed field across both the channel width and thickness.

The magnetic field strength was further characterized using the Hartmann number (Ha). The Ha number quantifies the relative influence of electromagnetic forces on the flow. The calculated Ha numbers were 1.06 for the cylindrical magnet and 1.42 for the rectangular magnet. These results confirm that the rectangular magnet exerts a stronger electromagnetic effect under otherwise identical operating conditions.

**Figure 6:**
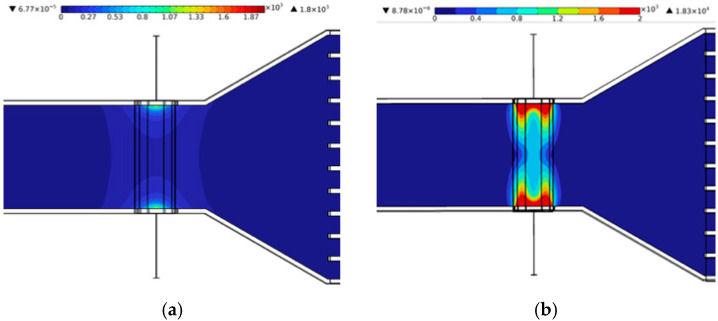
Current density distributions: (**a**) cylindrical magnet and (**b**) rectangular magnet.

**Figure 7:**
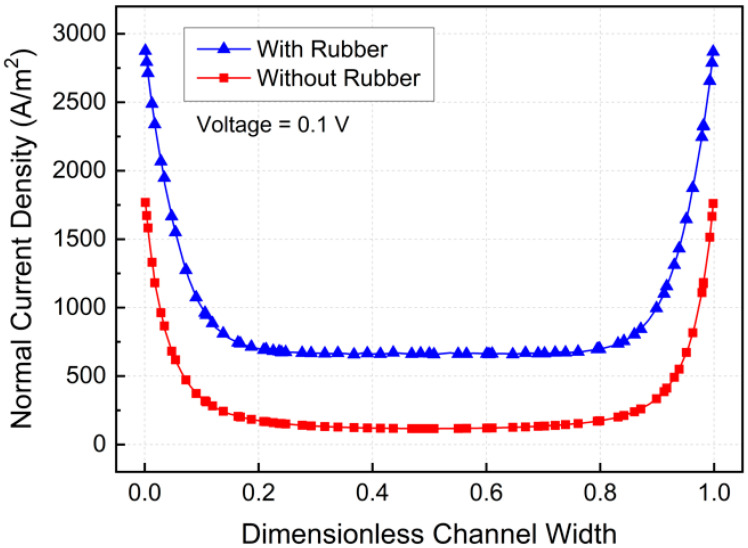
Normal current density variation with dimensionless width.

Figure 6 and Figure 7 present the distributions of normal current density for cases with and without rubber electrical insulation at an applied voltage of 0.1 V. In all cases, the current density was intensified near the channel walls due to electrode placement and electrical boundary conditions. When rubber insulation was not applied, part of the current leaked into the aluminum channel walls, reducing the effective current passing through the fluid. In contrast, the insulated configuration suppressed electrical leakage and forced a larger fraction of the current to flow through the ferrofluid. As a result, the average normal current density along the channel thickness direction increased from 280.43 A/m^2^ without insulation to 903.88 A/m^2^ with insulation, demonstrating a substantial enhancement of up to 222.31%.

**Figure 8:**
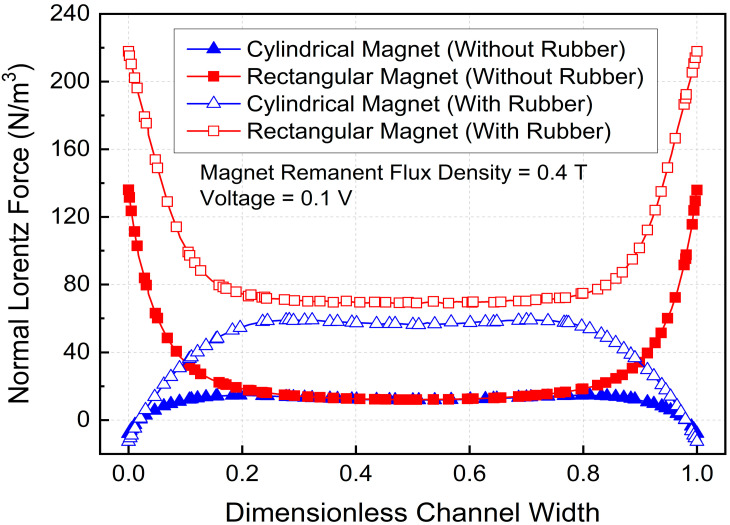
Normal Lorentz force variation with dimensionless width.

Because the Lorentz force is proportional to the cross product of the current density and the magnetic flux density, the combined distributions of these two fields directly determine the MHD system’s pumping capability. Figure 8 illustrates the normal Lorentz force distributions for different magnet geometries and insulation conditions. For the rectangular magnet, the magnetic field extended closer to the channel walls, allowing strong interaction with the wall-intensified current density and resulting in larger Lorentz forces distributed across the channel thickness. In contrast, the cylindrical magnet produces a Lorentz force distribution that is more concentrated near the channel center due to limited magnetic field penetration toward the walls.

The averaged normal Lorentz forces were 11.6 N/m^3^ and 46.7 N/m^3^ for the cylindrical magnet without and with rubber insulation, respectively, and 26.9 N/m^3^ and 89.7 N/m^3^ for the rectangular magnet. The application of rubber insulation increased the Lorentz force by 302.6% for the cylindrical magnet and 233.5% for the rectangular magnet. As a result, the weakest electromagnetic forcing was observed for the non-insulated cylindrical magnet, while the strongest was observed for the insulated rectangular magnet. These results clearly demonstrate that magnet geometry and electrical insulation play critical roles in enhancing electromagnetic forcing and MHD pump performance. The observed increase in electromagnetic forcing can be explained by the coupling between the electric current density and the magnetic flux density through the Lorentz force relation,${\vec{F}}_{L}=\vec{J}\times \vec{B}$. As the applied voltage increases, the electric field strength increases, raising the current density in the ferrofluid and thereby amplifying the Lorentz force. This trend is consistent with previous MHD pump studies, which reported that stronger electromagnetic forcing leads to larger body-force generation and improved pumping capability. In the present study, the rectangular magnet provides a wider magnetic field distribution across the channel thickness than the cylindrical magnet, enabling stronger interaction with the near-wall current and producing a higher average Lorentz force. This interpretation is consistent with previous studies showing that magnetic field distribution, rather than only peak field magnitude, strongly governs MHD pumping performance [37,38].

It should be noted that ferrofluids may exhibit a magnetoviscous effect in which the apparent viscosity increases when subjected to strong magnetic fields due to the alignment and interaction of magnetic nanoparticles. Such an increase in viscosity can potentially increase flow resistance and reduce the velocity induced by Lorentz forcing. However, in practical MHD cooling systems operating under moderate magnetic field strengths, the increase in viscosity is typically limited compared with the electromagnetic body force driving the flow. In addition, several engineering approaches are commonly adopted to mitigate the adverse effects of magnetically induced viscosity increases, including optimizing nanoparticle volume fraction, employing surfactant-stabilized ferrofluids to suppress particle chain formation, controlling the spatial distribution of magnetic fields, and optimizing channel geometry to reduce viscous losses. These considerations should be taken into account in the practical design of ferrofluid-based MHD cooling systems.

### 3.3. Flow Characteristics of MHD Pump

**Figure 9:**
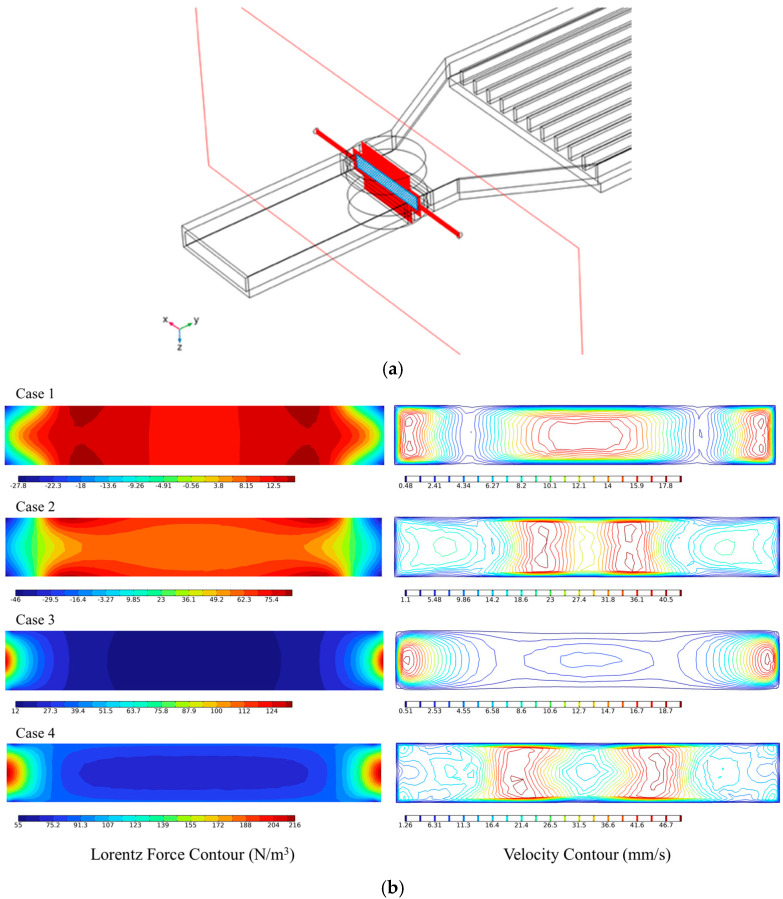
Lorentz force and velocity distributions with the section plane in the MHD acceleration region. (**a**) Location of the section plane. (**b**) Distributions of Lorentz force and velocity on the section plane.

This section examines the flow characteristics induced by Lorentz forcing in the MHD pump, focusing on the effects of magnet geometry and electrical insulation. Figure 9 illustrates the distributions of Lorentz force and velocity on a representative cross-sectional plane located within the electromagnetic acceleration region, as indicated in Figure 9a.

As shown in Figure 9b, when rubber insulation was not applied, part of the electric current leaks into the conductive channel walls, resulting in a relatively diffuse Lorentz force distribution and limited flow acceleration. In contrast, the insulated configuration confines the electric current within the fluid domain, leading to a substantially intensified Lorentz force and a pronounced increase in velocity. The velocity enhancement was most evident in regions away from the wall, demonstrating that localized Lorentz forcing directly governs the spatial acceleration of the flow.

Distinct differences in flow behavior were also observed between the two magnet geometries. Under identical remanent flux density and geometric constraints, the rectangular magnet produced a stronger, more extended magnetic field across the channel thickness, allowing the Lorentz force to act closer to the channel walls. As a result, the flow acceleration region extended toward the wall-adjacent zones, producing higher velocity levels than in the cylindrical magnet, which generated a more centrally concentrated Lorentz force and correspondingly more localized flow acceleration.

**Figure 10:**
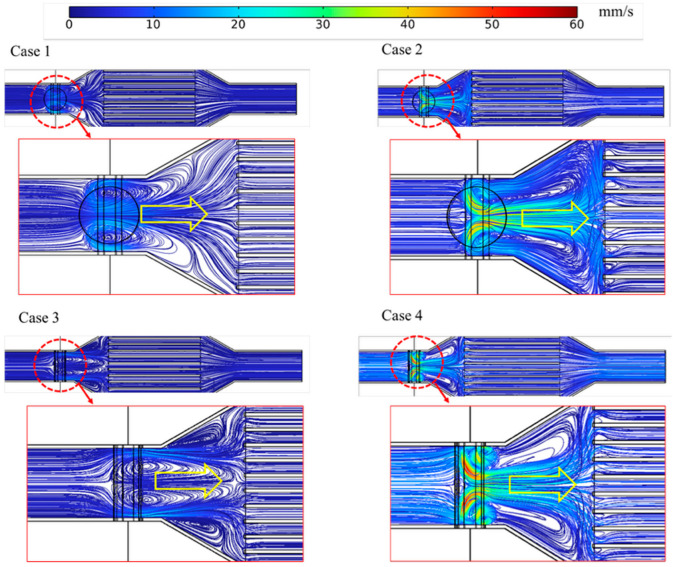
Streamline of the fluid for different magnet geometries and insulation conditions.

These characteristics are further clarified by the streamline patterns shown in Figure 10. In the rectangular magnet configuration, a higher streamline density was observed near the walls, indicating the formation of accelerated flow paths driven by enhanced electromagnetic forcing. In contrast, the cylindrical magnet exhibited more confined acceleration zones, while the channel core remained relatively uniform in flow for both cases. Overall, the streamline distributions were in good qualitative agreement with the Lorentz force and velocity fields shown in Figure 9, confirming the strong coupling between electromagnetic forcing and flow structure in the MHD pump.

The flow patterns confirm that the hydrodynamic response is governed by the spatial distribution of Lorentz forcing rather than by magnetic intensity alone. When electrical insulation is applied, current leakage into the conductive channel walls is suppressed, forcing a larger fraction of the current to flow through the ferrofluid. As a result, stronger local Lorentz forces are generated, leading to more effective flow acceleration. The rectangular magnet further enhances this effect because its magnetic field extends closer to the wall region, where the current density is also intensified. This produces stronger near-wall acceleration and higher wall shear, both of which are beneficial for disrupting the thermal boundary layer. Similar mechanisms have been reported in previous ferrofluid and MHD microchannel studies, where non-uniform magnetic forcing enhanced recirculation, mixing, and local wall convection [37].

### 3.4. Cooling Performance of MHD Microchannel Cooling System

**Figure 11:**
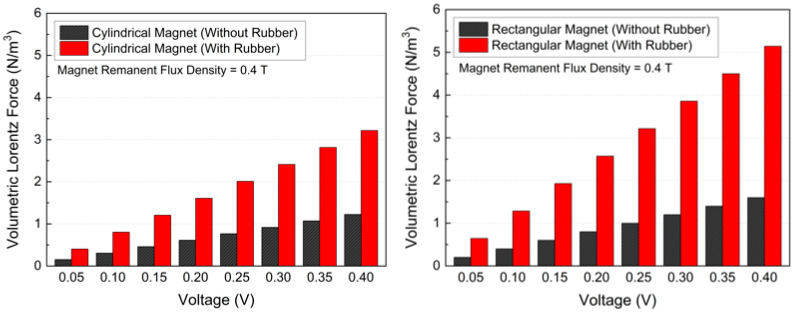
Variation of volumetric Lorentz force with various applied voltages in the MHD system.

The cooling performance of the MHD-assisted microchannel system was evaluated by examining the effects of applied voltage, magnet geometry, and electrical insulation on Lorentz force generation, flow acceleration, and battery thermal response. As the applied voltage increased from 0.05 V to 0.4 V, the normal current density increased approximately linearly, leading to a corresponding increase in the Lorentz force. This behavior results from the enhanced electric field strength, which drives a larger current through the ferrofluid and directly amplifies the electromagnetic body force. Figure 11 presents the variation of the volume-averaged Lorentz force density with applied voltage for different magnet geometries and insulation conditions. The reported values correspond to the electromagnetic body force averaged over the entire ferrofluid cooling channel domain. Figure 11 presents the variation of the Lorentz force with applied voltage for different magnet geometries and insulation conditions. For the cylindrical magnet, the Lorentz force increased from 0.1531 N/m^3^ to 1.22 N/m^3^ without rubber insulation, while a more pronounced increase from 0.4020 N/m^3^ to 3.2163 N/m^3^ was observed with insulation. A similar trend was obtained for the rectangular magnet, where the Lorentz force increased from 0.1998 N/m^3^ to 1.5983 N/m^3^ without insulation and from 0.6426 N/m^3^ to 5.1408 N/m^3^ with insulation. These results confirm that electrical insulation significantly enhances electromagnetic forcing by suppressing current leakage into the channel walls.

**Figure 12:**
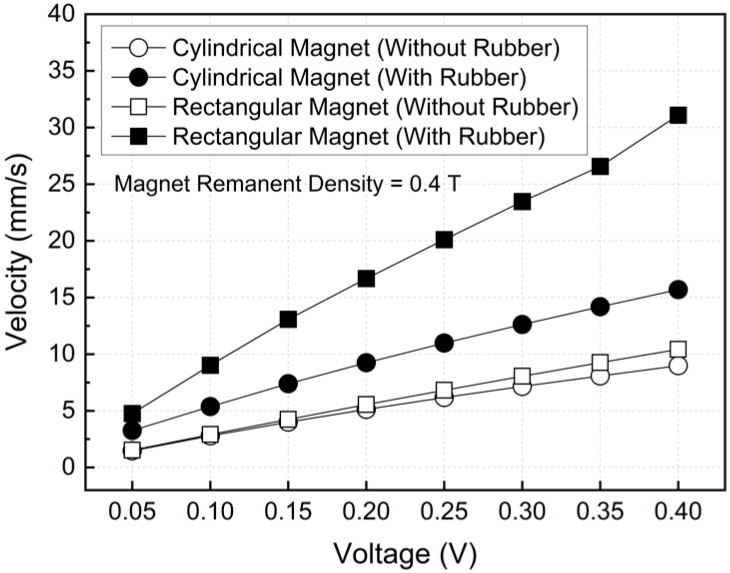
Variation of velocity with various applied voltages in the MHD system.

Figure 12 illustrates the variation of the average flow velocity with applied voltage for different magnet geometries and electrical boundary conditions. In all cases, the velocity increased almost linearly with voltage, which can be attributed to the corresponding increase in electric field strength, current density, and Lorentz force.

At a low applied voltage of 0.05 V, the cylindrical magnet without rubber insulation exhibited a very low average velocity of 1.5 mm/s, while the introduction of rubber insulation increased the velocity to 3.3 mm/s. Under the same voltage, the rectangular magnet showed a relatively higher velocity of 4.8 mm/s even without insulation, which further increased to 9.0 mm/s with insulation, indicating that the effect of magnetic field distribution clearly manifested even in the low-voltage regime.

As the applied voltage increased to 0.4 V, the differences among configurations became more pronounced. For the cylindrical magnet, the average velocity increased from 8.99 mm/s without insulation to 15.7 mm/s with insulation, whereas the rectangular magnet exhibited a much larger enhancement from 10.4 mm/s to 30.1 mm/s. These results demonstrate that the combined effects of electrical insulation, which suppresses current leakage and magnet geometry, provides a more uniform magnetic field distribution, significantly amplifying Lorentz-force-driven flow acceleration. Consequently, the configuration employing both a rectangular magnet and rubber insulation consistently achieved the highest velocities across the entire voltage range, confirming its superior MHD pumping performance.

**Figure 13:**
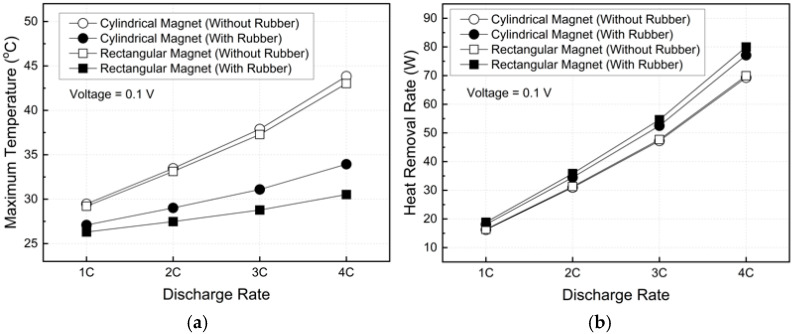
Cooling performance of the MHD system with various discharge rates: (**a**) maximum battery temperature and (**b**) heat removal rate.

Figure 13 presents the maximum battery temperature and heat removal rate as functions of the discharge rate under an applied voltage of 0.1 V. At 1C, the maximum battery temperature remained below approximately 29.46 °C for all cases, indicating stable operation regardless of magnet geometry or rubber insulation. However, at 4C, the configurations without rubber insulation exhibited a significant temperature rise, reaching 43.83 °C and 43.01 °C for the cylindrical and rectangular magnets, respectively, exceeding the commonly recommended upper limit of 40 °C for battery operation [51].

In contrast, with rubber insulation applied, the maximum battery temperature was effectively suppressed even at 4C, remaining at 33.94 °C for the cylindrical magnet and 30.53 °C for the rectangular magnet. Compared to the most unfavorable case, the maximum battery temperature was reduced by approximately 30.36%.

Despite these pronounced temperature differences, the heat removal rate varied only modestly among the cases. At 4C, the heat removal rate increased from about 69.3 W to 80.0 W, corresponding to a 13.38% increase. This limited variation results from a compensating mechanism between increased flow velocity and reduced fluid temperature rise (ΔT). Under steady-state conditions, forced convection dominates heat removal, and enhanced flow primarily reduces the battery surface temperature rather than significantly increasing the total heat removal rate.

**Figure 14:**
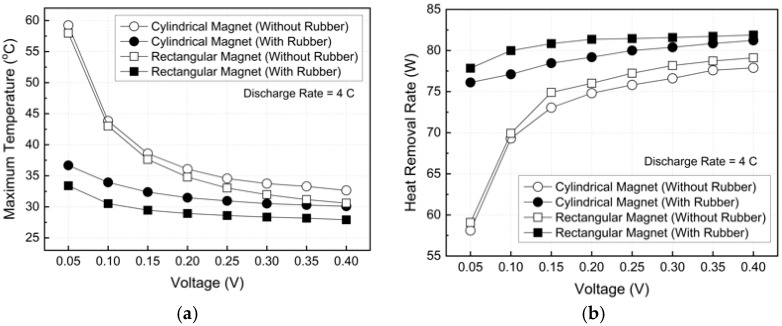
Cooling performance of the MHD system with various applied voltages: (**a**) maximum battery temperature and (**b**) heat removal rate.

Figure 14 compares the cooling performance at a 4C discharge rate under varying applied voltages. Without rubber insulation, the maximum battery temperature rose sharply in the low-voltage regime. At 0.05 V, the cylindrical and rectangular magnets exhibited maximum battery temperatures of 59.22 °C and 57.96 °C, respectively, both exceeding the recommended operating limit of 40 °C. Under these conditions, the corresponding heat removal rates were 58.1 W and 59.1 W, indicating weak electromagnetic forcing and extremely low flow velocities with a relatively significant contribution from natural convection.

With rubber insulation applied, the maximum battery temperature was substantially reduced at the same voltage. At 0.05 V, the temperature of the cylindrical magnet decreased from 59.22 °C to 36.68 °C, while that of the rectangular magnet decreased from 57.96 °C to 33.40 °C. As the applied voltage increased, the relative benefit of insulation diminished, and at 0.40 V, the maximum battery temperatures for the insulated cylindrical and rectangular magnets were reduced to 32.65 °C and 30.10 °C, respectively. At this voltage, the temperature reduction compared to the non-insulated case was limited to approximately 7.81%.

As shown in Figure 14b, the heat removal rate increased with increasing voltage for all cases, but the rate of increase gradually diminished. For example, the heat removal rate increased from 58.1 W at 0.05 V to 79.1 W and 81.2 W at 0.40 V for the insulated cylindrical and rectangular magnets, respectively. However, the incremental improvement became marginal when the voltage was increased from 0.35 V to 0.40 V, reflecting a diminishing sensitivity of mass flow rate, flow acceleration, and temperature difference (ΔT) to voltage.

**Figure 15:**
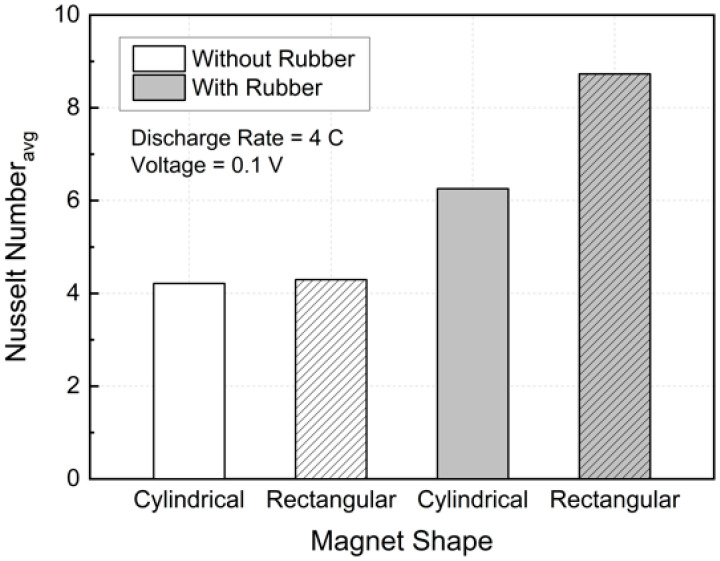
Variation of average Nusselt number for different magnet geometries and insulation conditions.

Figure 15 presents a quantitative comparison of convective heat transfer performance using the average Nusselt number at a 4C discharge rate and an applied voltage of 0.1 V. Without insulation, current leakage through the conductive channel walls resulted in weak and nearly uniform Lorentz forcing, leading to average Nusselt numbers of 4.21 and 4.30 for the cylindrical and rectangular magnets, respectively, with a marginal difference of approximately 2%.

When electrical insulation was applied, the confinement of current within the fluid domain significantly enhanced convective heat transfer. For the cylindrical magnet, the average Nusselt number increased from 4.21 to 6.25, corresponding to an enhancement of approximately 48.6%, whereas the rectangular magnet exhibited a much stronger increase from 4.30 to 8.73, representing an improvement of about 103%. The insulated rectangular magnet also achieved an average Nusselt number approximately 39.7% higher than that of the insulated cylindrical magnet.

This difference can be attributed to the magnetic field distribution associated with the magnet geometry. The rectangular magnet provides appreciable magnetic flux density toward the channel walls, enabling the confined near-wall current under insulated conditions to be effectively converted into strong Lorentz forcing. In contrast, the cylindrical magnet primarily concentrates the magnetic field in the core region, limiting near-wall forcing. Consequently, the rectangular magnet induces stronger near-wall shear and more effective thermal boundary layer disruption, resulting in superior convective heat transfer enhancement.

The thermal results indicate that the main benefit of enhanced MHD pumping is not only an increase in the total heat removal rate, but also a reduction in thermal resistance between the battery surface and the coolant. As the flow velocity increases, the thermal boundary layer becomes thinner and the wall shear stress increases, leading to stronger local convection and a larger Nusselt number. This explains why the insulated rectangular magnet case achieved the lowest battery temperature and the highest average Nusselt number. At higher applied voltages, however, the incremental benefit decreased because once the flow reaches sufficiently strong forced convection conditions, further increases in the Lorentz force yield diminishing improvements in temperature reduction and heat removal. This saturation behavior is consistent with prior MHD cooling studies, which also reported that stronger electromagnetic forcing improves cooling performance, but with progressively smaller gains at higher operating intensity [37,38].

## 4. Conclusions

This study numerically investigated the effects of electrical insulation and magnet geometry on the thermal performance of an MHD-driven microchannel cooling system for battery thermal management. In the absence of electrical insulation, current leakage through the conductive channel walls resulted in weak and nearly uniform Lorentz forcing, leading to limited differences in cooling performance between magnet geometries. In contrast, the application of electrical insulation effectively confined the electric current within the fluid domain, significantly enhancing both the reduction in maximum battery temperature and convective heat transfer performance.

Under a 4C discharge condition, the insulated rectangular magnet exhibited the highest thermal performance, achieving an increase of approximately 103% in the average Nusselt number compared to the non-insulated case and a 39.7% improvement relative to the insulated cylindrical magnet. This enhancement can be attributed to the magnetic field distribution of the rectangular magnet, which extended toward the channel walls and enabled the concentrated near-wall current under insulated conditions to be efficiently converted into strong Lorentz forcing, thereby intensifying wall shear and disrupting the thermal boundary layer.

Furthermore, the heat removal rate increased with applied voltage for all configurations; however, the rate of increase gradually diminished at higher voltages, indicating a saturation trend. This behavior suggests that once the mass flow rate becomes sufficiently large, further increases in voltage have a reduced influence on flow velocity and temperature difference, confirming that forced convection dominates the heat removal mechanism in the present system.

Overall, the results demonstrate that a proper combination of electrical insulation and optimized magnet geometry effectively transforms increased current density into localized near-wall Lorentz forcing, leading to a minimum enhancement of 48.6% in convective heat transfer performance. These findings provide practical design guidelines for the development of MHD-assisted active cooling systems in high-power battery and power electronic applications.

## Figures and Tables

**Table 1:** MHD system specifications.

Components		Parameters	Values
Rubber		Length (mm)	10
Cooling Plate		Length × Width × Height (mm)	668 × 70 × 16
		Thickness (mm)	3
		Number of channels fins (ea)	10
Magnet	Cylindrical	Radius × Height (mm)	50 × 10
	Rectangular	Length × Width × Height (mm)	24.8 × 70 × 10
Battery		Length × Width × Height (mm)	205 × 157 × 7

**Table 2:** A detailed description of the different configurations of the MHD system in the current study.

Design Cases	Conditions
Case 1	Without rubber, Cylindrical Magnet
Case 2	With rubber, Cylindrical Magnet
Case 3	Without rubber, Rectangular Magnet
Case 4	With rubber, Rectangular Magnet

**Table 3:** Thermophysical and electrical properties used in the simulations [43,44].

Specifications	Fe_3_O_4_	Water	Aluminum	Rubber	Battery
Density (kg/m^3^)	5180	998.2	2700	1100	1940
Specific heat (J/kg·K)	670	4182	900	1900	1000
Thermal conductivity (W/m·K)	80	0.6	238	0.5	18.2
Dynamic viscosity (Pa·s)	-	0.001	-	-	-
Electrical conductivity (S/m)	25,000	0.05	3.774 × 10^7^	1 × 10^−12^	-

**Table 4:** Boundary conditions for the numerical simulation of the current study.

Specifications	Values			
Ambient temperature (°C)	25			
Inlet coolant temperature (°C)	25			
Magnet remanent flux density (T)	0.4			
Applied voltage (V)	0.05, 0.10, 0.15, 0.20, 0.25, 0.30, 0.35, 0.40			
Battery discharge rate (C-rate)	1	2	3	4
Volumetric heat generation rate (W/m^3^)	86,109	163,785	249,451	365,299

## Data Availability

The data presented in this study will be available on request to the corresponding author.
